# Supplementary material for: Determining the adsorption energies of small molecules with the intrinsic properties of adsorbates and substrates
Source: Nat Commun. 2020 Mar 5;11:1196. doi: 10.1038/s41467-020-14969-8 (PMC7058040; doi:10.1038/s41467-020-14969-8)
Supplement: Supplementary file 1 — Supplementary Information [file 41467_2020_14969_MOESM1_ESM.pdf]

Supplementary Information

Determining the adsorption energies of small molecules with the intrinsic properties of adsorbates and substrates

Wang Gao,* Yun Chen, Bo Li, Shan-Ping Liu, Xin Liu, and Qing Jiang*

Key Laboratory of Automobile Materials, Ministry of Education, and College of Materials Science and Engineering, Jilin University, Changchun 130022, China

*E-mail: wgao@jlu.edu.cn; jiangq@jlu.edu.cn.

Table of contents page

Supplementary Note 1	1
Supplementary Note 2	2
Supplementary Note 3	4
Supplementary Note 4	6
Supplementary Note 5	7
Supplementary Figure 1	8
Supplementary Figure 2	9
Supplementary Figure 3	10
Supplementary Figure 4	11
Supplementary Figure 5	12
Supplementary Figure 6	13
Supplementary Figure 7	14
Supplementary Figure 8	15
Supplementary Figure 9	16
Supplementary Figure 10	16
Supplementary Figure 11	17
Supplementary Figure 12	18
Supplementary Table 1	19
Supplementary Table 2	20
Supplementary Table 3	21
Supplementary Table 4	22
Supplementary Table 5	23
Supplementary Table 6	23
Supplementary Table 7	24
Supplementary Table 8	25
Supplementary Table 9	25
Supplementary Table 10	26
Supplementary Table 11	27
Supplementary Table 12	28
Supplementary Table 13	28
Supplementary Table 14	29
Supplementary Table 15	29
Supplementary Table 16	30
Supplementary Table 17	31
Supplementary Table 18	33

Supplementary Note 1: Extension of our model for molecules with two kinds of functional groups

Equations (3), (5) - (7) and (9) in the main text are suitable for the hydrogenates and oxygenates with one kind of functional groups binding to the central atom. Actually, with regard to the central atoms with two kinds of functional groups such as CHO, COOH and CHOH, the coefficient α will change to a new form:

$$\alpha = \frac{X_\mathrm{m}-X}{X_\mathrm{m}+1} - \frac{X'}{X'_\mathrm{m}+1} \tag{1}$$

Where X' and X_m' are the actual bonding number and maximum bonding number binding to the central atom for the second functional group, respectively. Hence, the slope k of the linear relation for the hydrogenates and oxygenates with two kinds of functional groups will be as follows:

$$k = 0.1 \times \alpha = 0.1 \times \left[\frac{X_\mathrm{m}-X}{X_\mathrm{m}+1} - \frac{X'}{X'_\mathrm{m}+1}\right] \tag{2}$$

Apparently, a general expression for all adsorbates is $E_\mathrm{ad} = 0.1 \times \alpha \times \psi + 0.2 \times (1-\alpha) \times \overline{CN} + \theta$, in which if α is substituted with Supplementary Equation 1, one obtains that:

$$\begin{aligned} E_\mathrm{ad} &= 0.1\times\alpha\times\psi + 0.2\times(1-\alpha)\times\overline{CN} + \theta \\ &= 0.1\times\left[\frac{X_\mathrm{m}-X}{X_\mathrm{m}+1} - \frac{X'}{X'_\mathrm{m}+1}\right]\times\psi + 0.2\times\left[\frac{X+1}{X_\mathrm{m}+1} + \frac{X'}{X'_\mathrm{m}+1}\right]\times\overline{CN} + \theta \end{aligned} \tag{3}$$

Meanwhile, the adsorption energy difference between any pair of adsorbates with the same central atom for two functional groups is given as:

$$\begin{aligned} \Delta E_\mathrm{ad} &= 0.1\times(\alpha_1-\alpha_2)\times\psi + 0.2\times(\alpha_2-\alpha_1)\times\overline{CN} + \theta'_{1,2} \\ &= 0.1\times\left[\frac{X_2-X_1}{X_\mathrm{m}+1} - \frac{X'_1-X'_2}{X'_\mathrm{m}+1}\right]\times\psi + 0.2\times\left[\frac{X_1-X_2}{X_\mathrm{m}+1} - \frac{X'_2-X'_1}{X'_\mathrm{m}+1}\right]\times\overline{CN} + \theta'_{1,2} \end{aligned} \tag{4}$$

Obviously, the adsorption energies of adsorbates with one or two kinds of functional groups follow the same rule.

Supplementary Note 2: The derivation of LSRs and its generalized form

We will first use our model to derive the linear scaling relationships (LSRs) established by Nørskov *et al.*[1] and its generalized form established by Calle-Vallejo *et al.*[2]. On the basis of Equations (2) and (3) in the main text, the adsorption energies are given as:

$$\begin{aligned} E_{\mathrm{ad}} &= k\psi + b \\ &= 0.1\times\alpha\times\psi + b \end{aligned} \tag{5}$$

Note that the actual bonding number X for an atom is zero (X = 0), therefore, the adsorption energy of an atom with Supplementary Equation 5 can be reorganized to give:

$$\begin{aligned} E_{\mathrm{ad}}^{\mathrm{A}} &= k^{\mathrm{A}}\psi + b^{\mathrm{A}} \\ &= 0.1\times\alpha^{\mathrm{A}}\times\psi + b^{\mathrm{A}} \\ &= 0.1\times\frac{X_{\mathrm{m}}}{X_{\mathrm{m}}+1}\times\psi + b^{\mathrm{A}} \end{aligned} \tag{6}$$

For the atoms' partially hydrogenated species, the adsorption energies are as follows:

$$\begin{aligned} E_{\mathrm{ad}}^{\mathrm{AH}_x} &= k^{\mathrm{AH}_x}\psi + b^{\mathrm{AH}_x} \\ &= 0.1\times\alpha^{\mathrm{AH}_x}\times\psi + b^{\mathrm{AH}_x} \\ &= 0.1\times\frac{X_{\mathrm{m}}-X}{X_{\mathrm{m}}+1}\times\psi + b^{\mathrm{AH}_x} \end{aligned} \tag{7}$$

Comparing Supplementary Equations 6 with 7, we can naturally derive that:

$$\begin{aligned} E_{\mathrm{ad}}^{\mathrm{AH}_x} &= \frac{\alpha^{\mathrm{AH}_x}}{\alpha^{\mathrm{A}}}\times E_{\mathrm{ad}}^{\mathrm{A}} + \left[b^{\mathrm{AH}_x} - \frac{\alpha^{\mathrm{AH}_x}}{\alpha^{\mathrm{A}}}\times b^{\mathrm{A}}\right] \\ &= \frac{X_{\mathrm{m}}-X}{X_{\mathrm{m}}}\times E_{\mathrm{ad}}^{\mathrm{A}} + \left[b^{\mathrm{AH}_x} - \frac{X_{\mathrm{m}}-X}{X_{\mathrm{m}}}\times b^{\mathrm{A}}\right] \\ &= \gamma E_{\mathrm{ad}}^{\mathrm{A}} + \left(b^{\mathrm{AH}_x} - \gamma b^{\mathrm{A}}\right) \\ &= \gamma E_{\mathrm{ad}}^{\mathrm{A}} + \xi \end{aligned} \tag{8}$$

This expression is exactly the LSRs.

For the generalized LSRs form established by Calle-Vallejo *et al.*[2], the offset ξ scales with coordination number for any pair of adsorbates (with X_1 and X_2) with the same central atom, that is:

$$\xi = \mu\overline{CN} + \theta_{1,2} \tag{9}$$

With Supplementary Equations 8 and 9, we obtain:

$$\xi = \mu\overline{CN} + \theta_{1,2} = b^{\mathrm{AH}_{x_2}} - \gamma b^{\mathrm{AH}_{x_1}} \tag{10}$$

Recalling Equations (4) and (5) in the main text for calculating b,

$$b = 0.2\times(1-\alpha)\overline{CN} + \theta = 0.2\times\frac{X+1}{X_{\mathrm{m}}+1}\overline{CN} + \theta \tag{11}$$

Now, one can derive the coordination dependence of the offset ξ of the generalized LSRs, for any pair

of adsorbates (with X_1 and X_2) with the same central atom as,

$$\begin{aligned}\xi &= \mu\overline{CN} + \theta_{1,2} = b^{\mathrm{AH}_{x2}} - \gamma b^{\mathrm{AH}_{x1}} \\ &= 0.2\times(1-\alpha_2)\times\overline{CN} + \theta^{\mathrm{AH}_{x2}} - 0.2\times\frac{\alpha_2}{\alpha_1}\times(1-\alpha_1)\times\overline{CN} - \frac{\alpha_2}{\alpha_1}\times\theta^{\mathrm{AH}_{x1}} \\ &= 0.2\times\frac{\alpha_1-\alpha_2}{\alpha_1}\times\overline{CN} + \left[\theta^{\mathrm{AH}_{x2}} - \frac{\alpha_2}{\alpha_1}\times\theta^{\mathrm{AH}_{x1}}\right] \\ &= 0.2\times\frac{X_2-X_1}{X_{\mathrm{m}}-X_1}\times\overline{CN} + \left[\theta^{\mathrm{AH}_{x2}} - \frac{X_{\mathrm{m}}-X_2}{X_{\mathrm{m}}-X_1}\times\theta^{\mathrm{AH}_{x1}}\right]\end{aligned} \tag{12}$$

Clearly, Supplementary Equation 12 is a generalized form of the offset ξ of LSRs. Note that the usual coordination number CN and the generalized coordination number $\overline{CN}$ are almost identical in describing transition-metal (TM) extended surfaces.

Supplementary Note 3: Extension of our model for intermetallics and oxides

For a given adsorption site, it exhibits significantly different electronic structures on near-surface alloys (NSAs) and oxides compared with pure transition metals (TMs) and nanoparticles (NPs) due to its coordination environments. To incorporate the local environment effect of active centers, the electronic descriptor ψ is generalized by using the geometric mean of the valence number S_v and electronegativity χ of the given substrate atoms and their neighboring atoms, as

$$\psi = \frac{(\prod_{i=1}^{N} S_{v_i})^{2/N}}{(\prod_{i=1}^{N} \chi_i)^{1/N}} \tag{13}$$

where N is the number of the atoms at active centers, while S_{vi} and χ_i are the outer-electron number and electronegativity of the ith atom at active centers. It is noteworthy that Supplementary Equation 13 is automatically converted into Equation (1) in the main text for calculating ψ in pure TMs and NPs, namely Supplementary Equation 13 is universal for TMs, NPs, NSAs, and oxides. Taking a top-site adsorption case as an example to elucidate the descriptor. On Pt-X@Pt(111), the local active centers contain one Pt atom at the adsorption site with $N_{\text{adsorption}}$ (Pt) = 1, and six Pt atoms with N_{1st} (Pt) = 6 and three X atom with N_{1st} (X) = 3 at the first-nearest neighboring sites (Supplementary Figure 9b). Whether to consider the effect of the second-nearest neighbors on the active center depends entirely on surface structures, which can be easily identified through the unit cells. As shown in Supplementary Figure 10, the effect of the second-nearest neighbors needs to be taken into account in dioxides (MO_2) and perovskite oxides (ABO_3). Accordingly, in the case of MO_2(110) the local active centers contain one M atom at the adsorption site with $N_{\text{adsorptoin}}$ (M) = 1, five first-nearest neighboring oxygens with N_{1st} (O) = 5, and one M atom at the second-nearest neighboring site with N_{2nd} (M) = 1. For ABO_3(100), the local active centers contain one B atom at the adsorption site with $N_{\text{adsorptoin}}$ (B) = 1, five first-nearest neighboring oxygens with N_{1st} (O) = 5, and one A atom at the second-nearest neighboring site with N_{2nd} (A) = 1 (see Supplementary Table 16). The small number of N_{2nd} (M) and N_{2nd} (A) is due to the share of the element among different active centers. Moreover, it has been demonstrated that the number of outer electrons for the elements in the second-nearest neighboring sites is secondary in determining the adsorption energy at the active centers of oxides[3], implying that the second-nearest neighboring elements likely function only via electronegativity. $S_{\text{v,2nd}}$ (M) for MO_2 and $S_{\text{v,2nd}}$ (A) for ABO_3 are thus excluded in Supplementary Equation 13. Consequently, one obtains ψ in Supplementary Table 13 and its scaling with adsorption energy in Figure 1m-o in the main text. Note that our model also identifies that the element A of ABO_3 plays a secondary role in

determining the adsorption energies of B sites: the exclusion of the effect of element A only has a minor effect on the scaling between ψ and adsorption energy as shown in Supplementary Figure 11.

Supplementary Note 4: Correlation with the established model

To elucidate the connection between d-band model and our electronic descriptor ψ, we plot the d-band center ε_d by the NA model as a function of ψ as shown in Fig. 3a in the main text[4], finding a clear linear relationship between ε_d and ψ for 17 TMs in all considered 24 TMs, with the other 7 TMs as outliers. By introducing the W_d effect on the top of ε_d with the formula of $\varepsilon_d+W_d/8$ for the 7 outlying metals, these 7 TMs immediately move to the queue established for the other 17 TMs that only uses ε_d (Supplementary Figure 12a). By introducing the W_d effect on the top of ε_d with the formula of $\varepsilon_d-W_d/8$ for the 17 TMs (but only using ε_d for 7 outlying metals), the 24 TMs consistently display a quadratic relationship on ψ (Supplementary Figure 12b). It is noteworthy that a universal linear relationship between the descriptor ψ and the proposed generalized descriptor $\varepsilon_d+W_d/2$ has also been found for all considered 24 TMs as shown in Fig. 3b in the main text. For adsorption on transition metals, the bond strength is given by the position and filling of the anti-bonding states, which are pinned at the upper edge of the d-band (namely $\varepsilon_d+W_d/2$). The descriptor, $\varepsilon_d+W_d/2$, accounts for the effects of both the average energy of d-bands and their spread energy on the position of adsorbate-metal antibonding states. However, the descriptor by the NA model, to the best of our knowledge, is mainly applied to pure TMs with few intermetallics[4], but not into oxides. Besides TMs and NPs, we have generalized our model into NSAs and oxides, in particular for the adsorption of OH, F and Cl on NSAs and the adsorption of OH_x on oxides that cannot be accurately described by the d-band model [3,5-7].

Supplementary Note 5: The gas-phase references for adsorption energies

The absolute values of adsorption energies are distinct depending on the adopted energy references for small molecules (see Supplementary Table 17 and references therein). To ensure the comparability of the cited data, we unified the energy references for the cited data by shifting the energy difference between the standard references that we choose and the original ones. In the case of molecular species binding with C terminal, we chose the gaseous CO, H_2O and H_2 as the references[8]. For the molecular species binding with N terminal, N_2 and H_2 were taken as the references. In addition, the gas-phase references for the adsorption energies of *O, *OH, *OOH and *OCH_3 were 1/2O_2, OH, OOH and OCH_3, respectively (see Supplementary Table 18). Consequently, the internal consistency and comparability have been found for the cited data, ensuring the validity of our model well.

All DFT calculations for the gas-phase references were performed by the spin-polarized PBE[9] exchange-correlation functional, implemented with the Vienna Ab Initio Simulation Package (VASP)[10]. A primitive cubic cell of 25 Å × 25 Å × 25 Å was used with a 1 × 1 × 1 k-grid centered at the gamma (Γ) point for the Brillouin zone. The cutoff energy of plane wave was set to 400 eV; the energy convergence criteria of optimization was 10^{-6} eV/atom, and the force convergence criteria of optimization was 10^{-2} eV/Å.

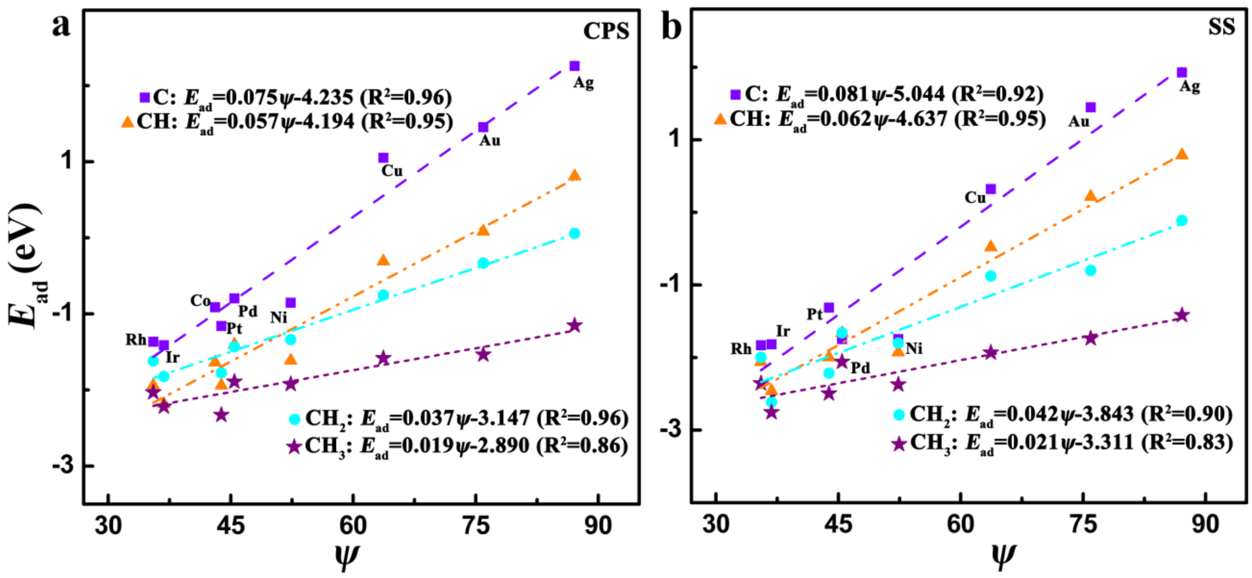

Supplementary Figure 1 | Adsorption energies of CH_x (x=0, 1, 2, 3) versus the electronic descriptor ψ on transition-metal (TM) surfaces. (a) CH_x (x=0, 1, 2, 3) on close-packed TM surfaces (CPS). (b) CH_x (x=0, 1, 2, 3) on stepped TM surfaces (SS). In each panel, the adsorption energies of adsorbates are linearly correlated with ψ. All data are calculated by using the RPBE functional[1,11]. Source data are provided as a Source Data file.

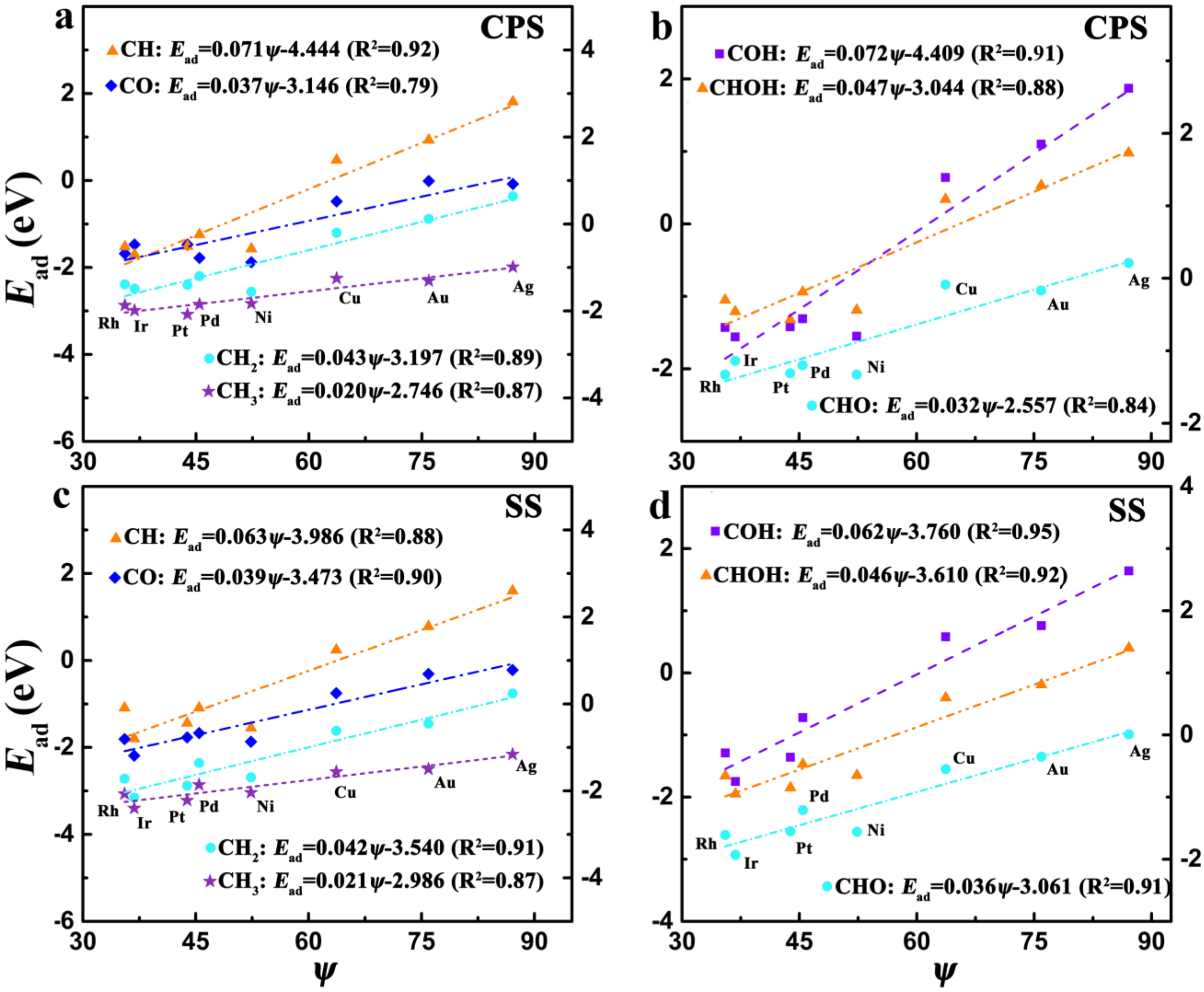

Supplementary Figure 2 | Adsorption energies of C-terminated molecules versus the electronic descriptor ψ on transition-metal (TM) surfaces. (a) [(c)] CO and CH_x (x=1, 2, 3) on TM close-packed surfaces (CPS) [stepped surfaces (SS)]. (b) [(d)] COH, CHOH and CHO on close-packed surfaces (CPS) [stepped surfaces (SS)]. In each panel, the adsorption energies of adsorbates are linearly correlated with ψ. All data are calculated by using the BEEF-vdW functional[12,13]. In each subfigure with both left and right axes, the linear fits at the upper left corner correspond to the left coordinate axis and those at the bottom right corner correspond to the right coordinate axis. Source data are provided as a Source Data file.

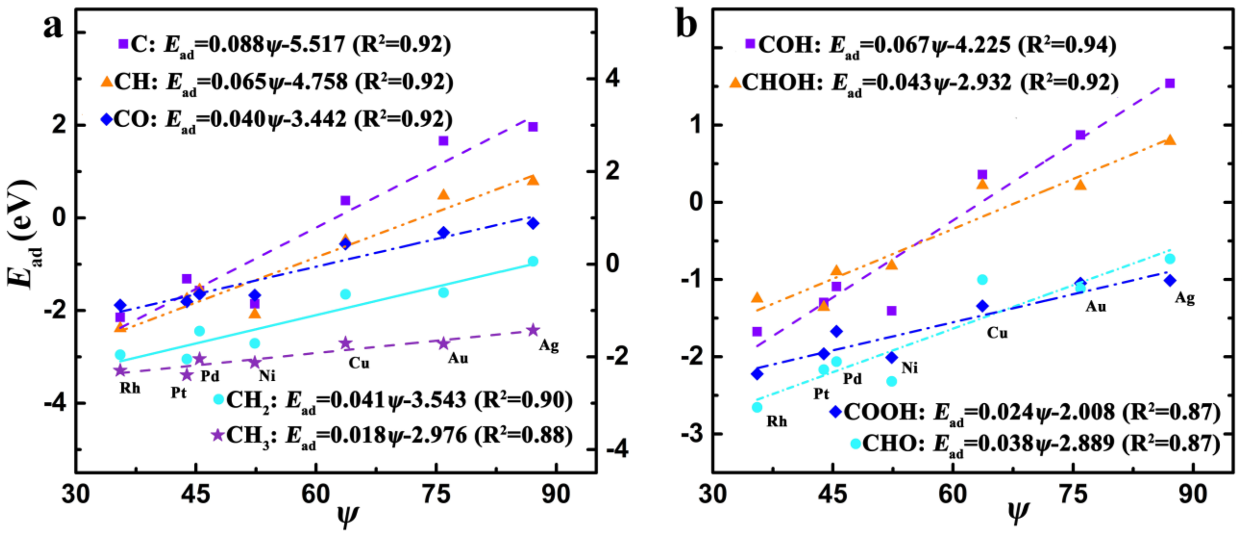

Supplementary Figure 3 | Adsorption energies of C-terminated molecules versus the electronic descriptor *ψ* on transition-metal (TM) (100) surfaces. (a) CO and CH_x (x=0, 1, 2, 3) on TM (100) surfaces. (b) COH, CHOH, COOH and CHO on TM (100) surfaces. In each panel, the adsorption energies of adsorbates are linearly correlated with *ψ*. All data are calculated by using the RPBE functional[14]. In each subfigure with both left and right axes, the linear fits at the upper left corner correspond to the left coordinate axis and those at the bottom right corner correspond to the right coordinate axis. Source data are provided as a Source Data file.

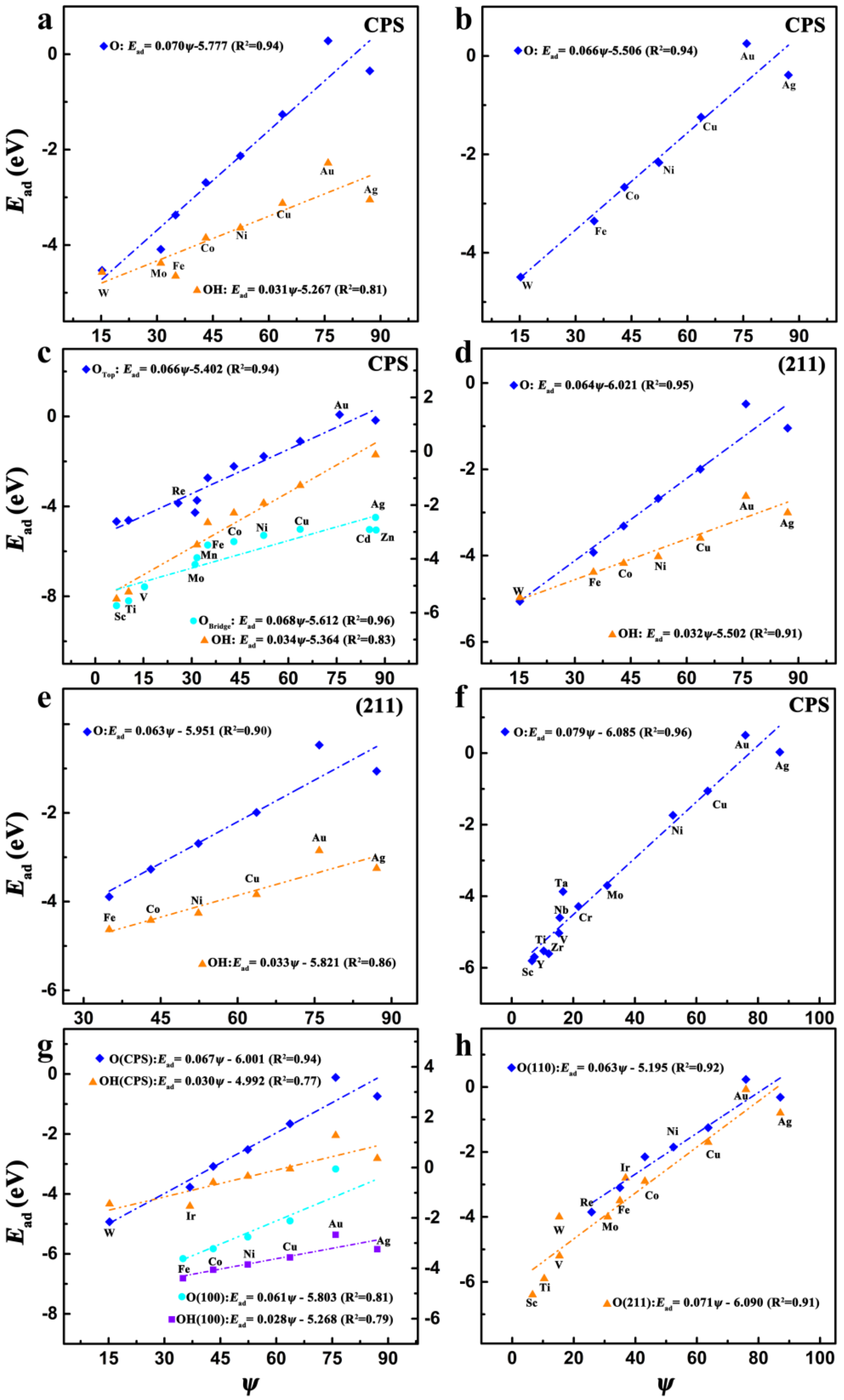

Supplementary Figure 4 | Adsorption energies of O and OH versus the electronic descriptor *ψ* on transition-metal (TM) extended surfaces. The data for O and OH on TM close-packed surfaces (CPS) is shown in (a), (b), (c), (f) [including O only] and (g), while the data for O and OH on TM stepped surfaces is shown in (d), (e), (g) and (h) [including O only]. In each panel, the adsorption energies of adsorbates are linearly correlated with *ψ*. All data are calculated by using the RPBE functional[1,4,5,15-19]. In each subfigure with both left and right axes, the linear fits at the upper left corner correspond to the left coordinate axis and those at the bottom right corner correspond to the right coordinate axis. Source data are provided as a Source Data file.

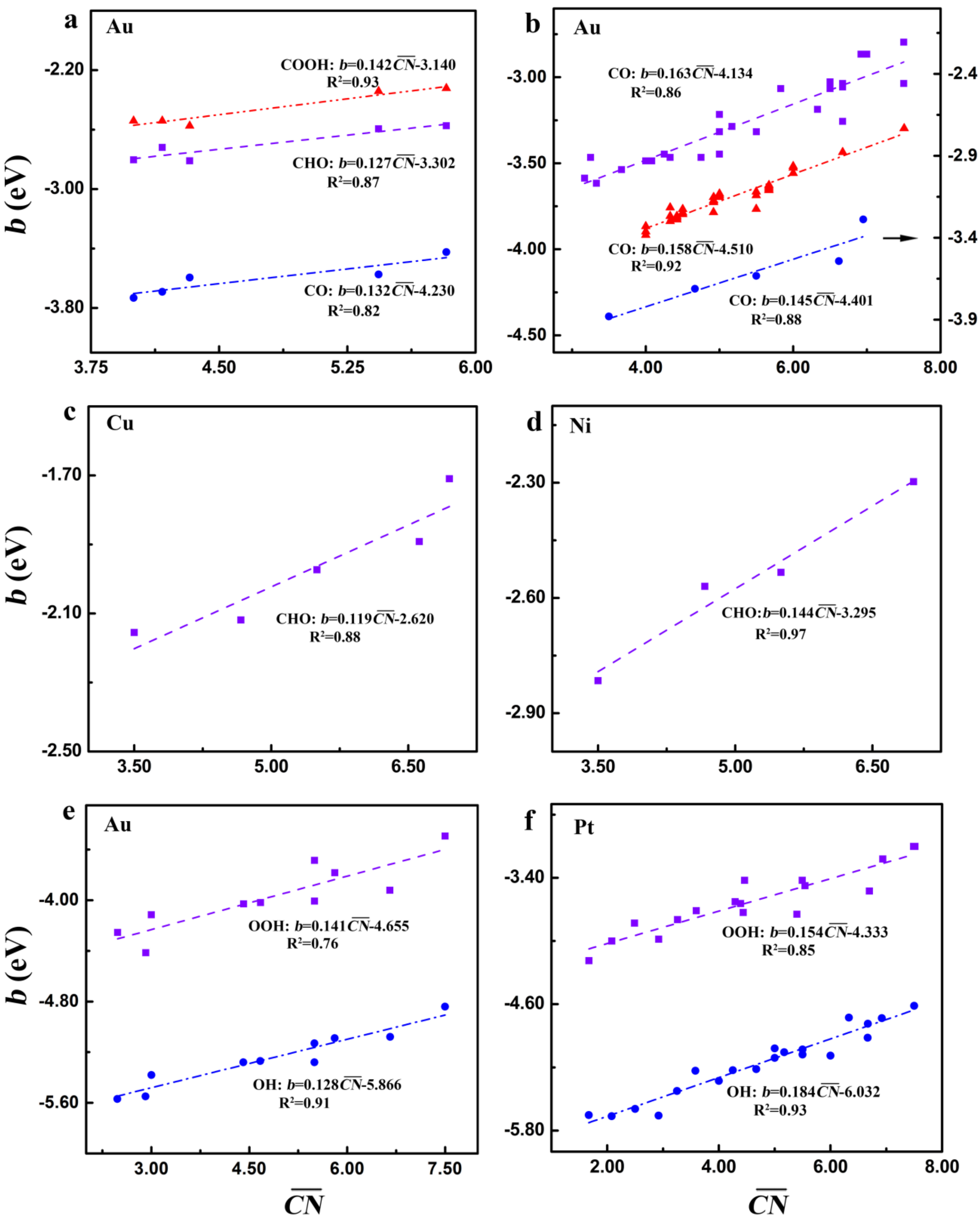

Supplementary Figure 5 | Structure-offset *b* relations on transition-metal (TM) surfaces and nanoparticles. (a) CO, CHO and COOH on Au surfaces. (b) CO on Au surfaces and nanoparticles. (c) [(d)] CHO on Cu (Ni) surfaces. (e)[(f)] OH and OOH on Au surfaces (Pt surfaces and nanoparticles). In each panel, the offset b of the ψ-determined scaling relation is linearly correlated with the generalized coordination number ($\overline{CN}$) of surfaces. All data are calculated by using (a) RPBE and (b) - (f) PBE functionals[20-25]. Source data are provided as a Source Data file.

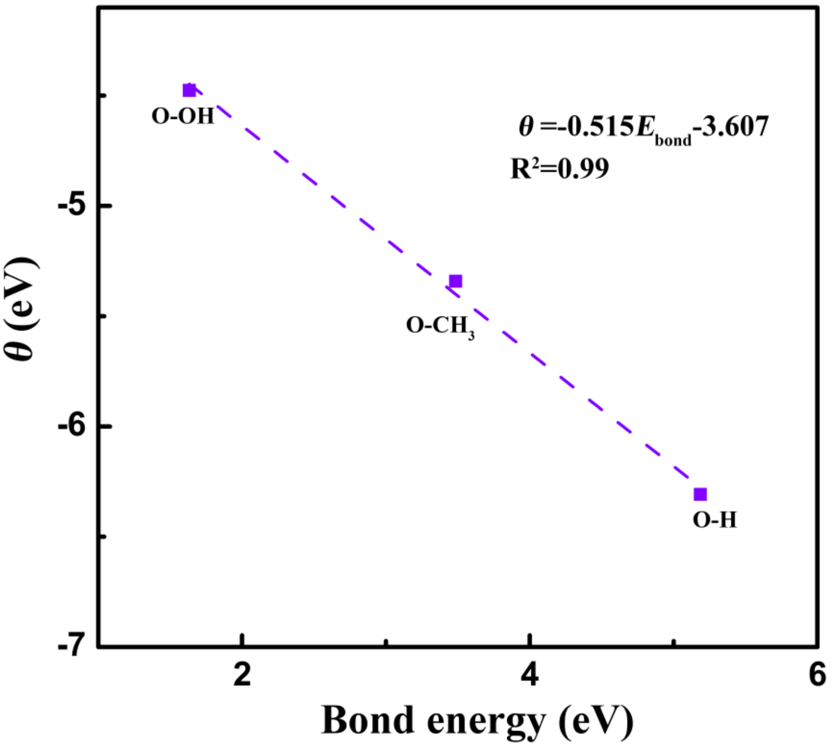

Supplementary Figure 6 | The relation between θ and bond energy. The θ (of Equation (7) in the main text) of the species binding via oxygen as a function of the bond energy between the oxygen atom and its coordination atoms in adsorbates (O-O bond for OOH, O-C bond for OCH_3 and O-H bond for OH). Apparently, θ strongly depends on the bond energy between oxygen and its coordination atoms in adsorbates. Source data are provided as a Source Data file.

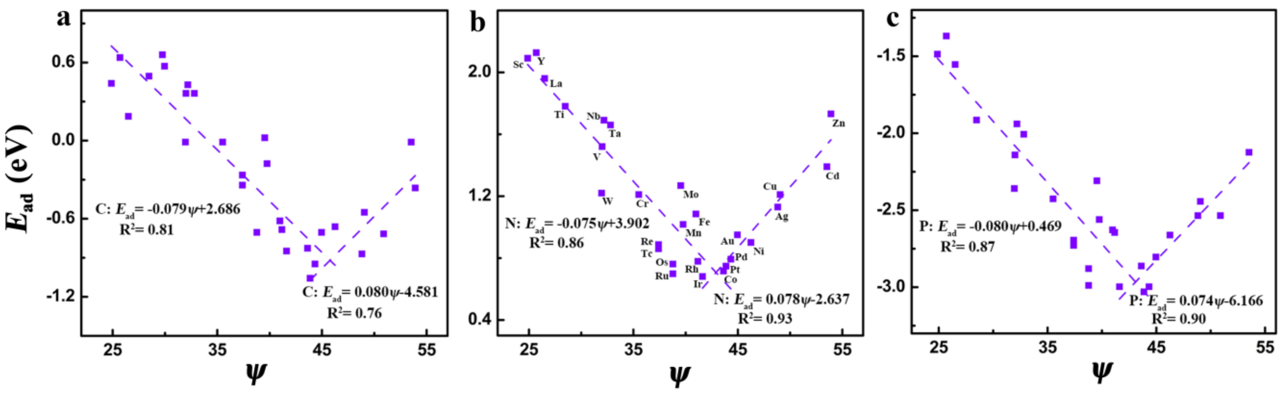

Supplementary Figure 7 | Adsorption energies of single atoms versus the electronic descriptor *ψ* on near-surface alloys (NSAs) surfaces of Pt. The adsorption energies of (a) C, (b) N and (c) P single atoms versus *ψ* on (111) surfaces of Pt-NSAs. All data are calculated by using the PBE functional[26]. Source data are provided as a Source Data file.

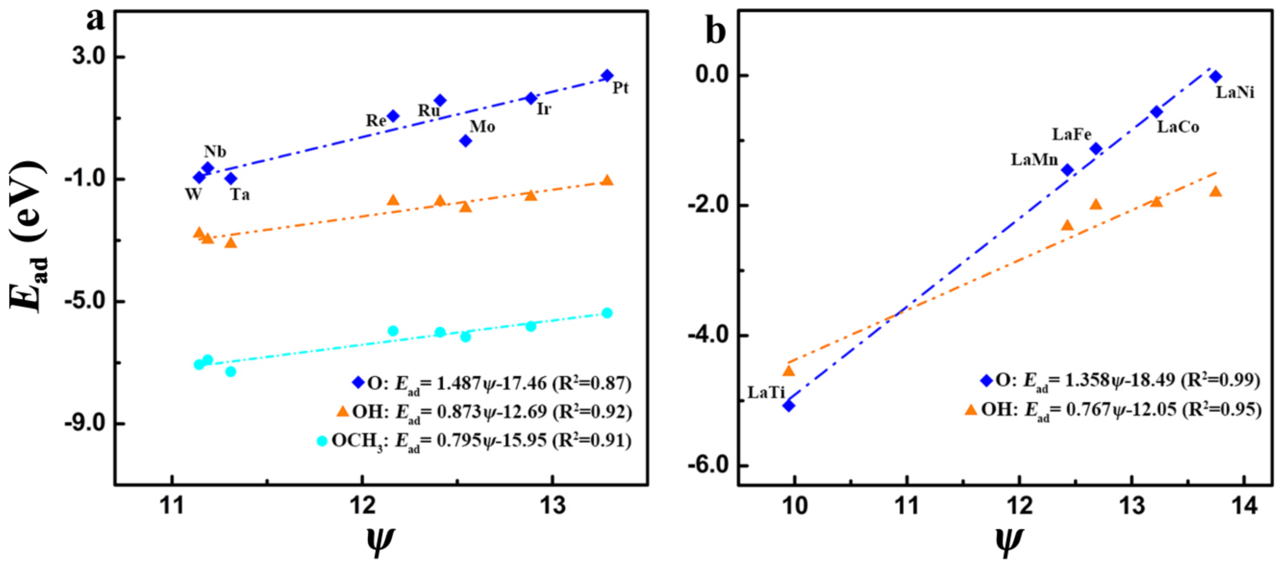

Supplementary Figure 8 | Adsorption energies of O-terminated molecules versus the electronic descriptor ψ on oxide surfaces. (a) The adsorption energies of O, OH and OCH_3 versus ψ at transition-metal (TM) oxide overlayer on RuO_2 (110) surfaces. All data are calculated by using the BEEF-vdW functional[27]. (b) the adsorption energies of O and OH versus ψ on (100) surfaces of perovskite oxides ABO_3. In each panel, the adsorption energies of adsorbates are linearly correlated with ψ. All data are calculated by using the RPBE functional[16]. Source data are provided as a Source Data file.

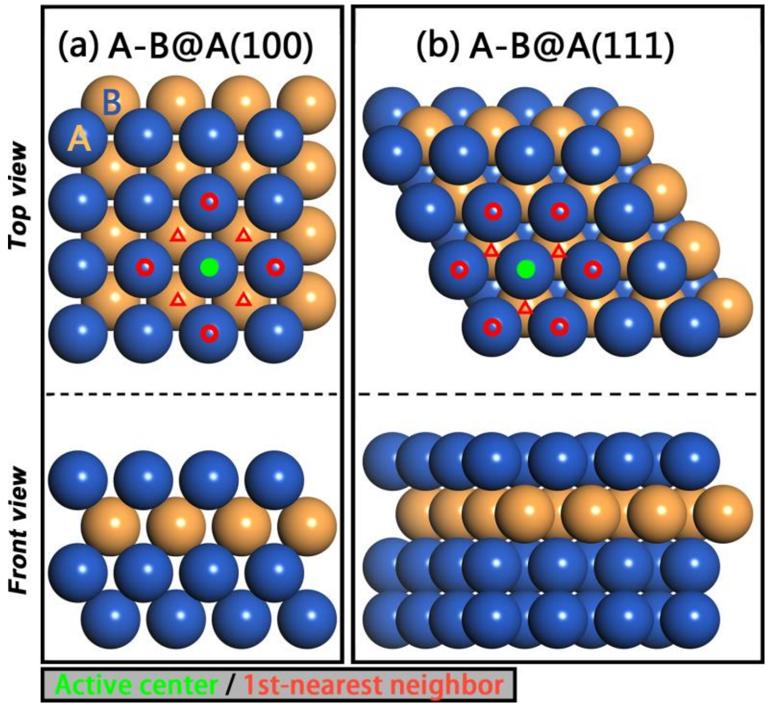

Supplementary Figure 9 | Schematic illustrations of the active centers on near-surface alloys (NSAs). (a) Active centers on (100) surface of A-B@A [A-B@A (100)]. (b) Active centers on (111) surface of A-B@A [A-B@A(111)]. Circles and triangles correspond to A and B atoms in the NSAs, while filled and empty labels denote the active central site and its first-nearest neighboring sites.

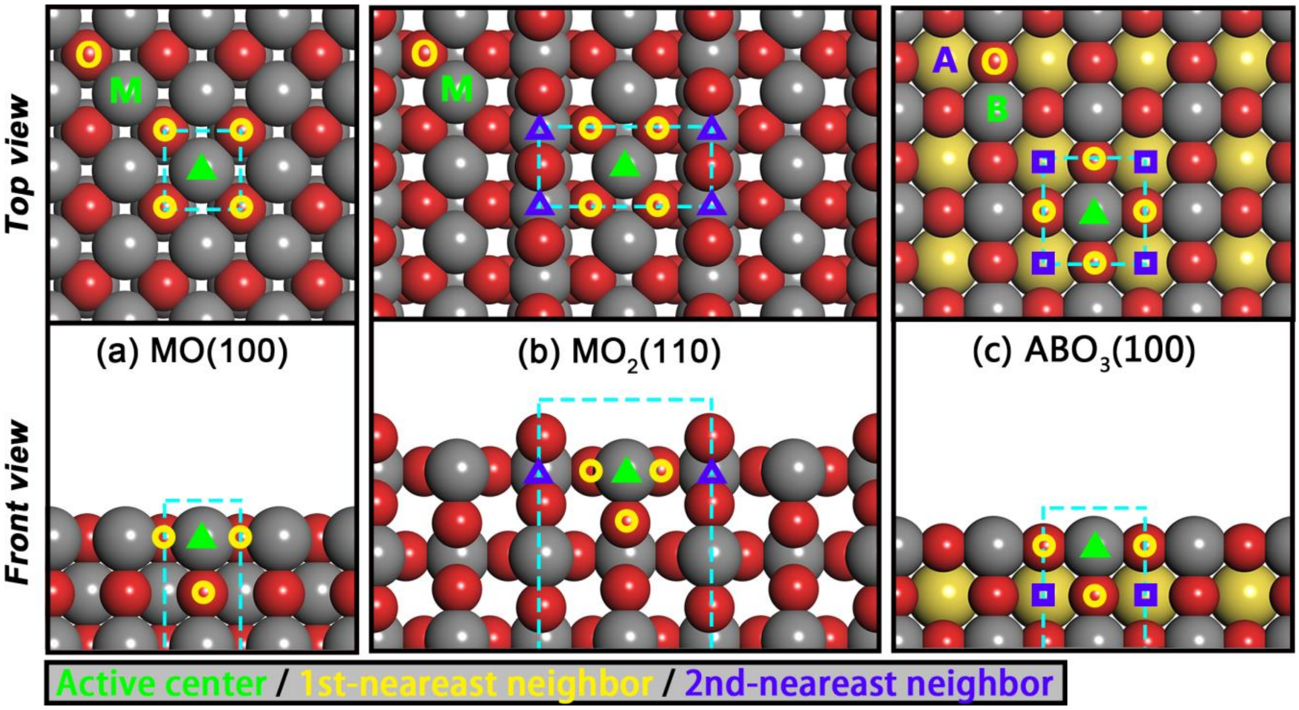

Supplementary Figure 10 | Schematic illustrations of the active centers on oxides. (a) Active centers on (100) surface of monoxides MO(100). (b) Active centers on (110) surface of dioxides MO_2(110). (c) Active centers on (100) surface of perovskite oxides ABO_3(100). Triangles, circles and squares correspond to the different atomic species, while filled triangles denote the active central sites, empty circles correspond to the first-nearest neighboring sites, and empty triangles and squares represent the second-nearest neighboring sites in a unit cell.

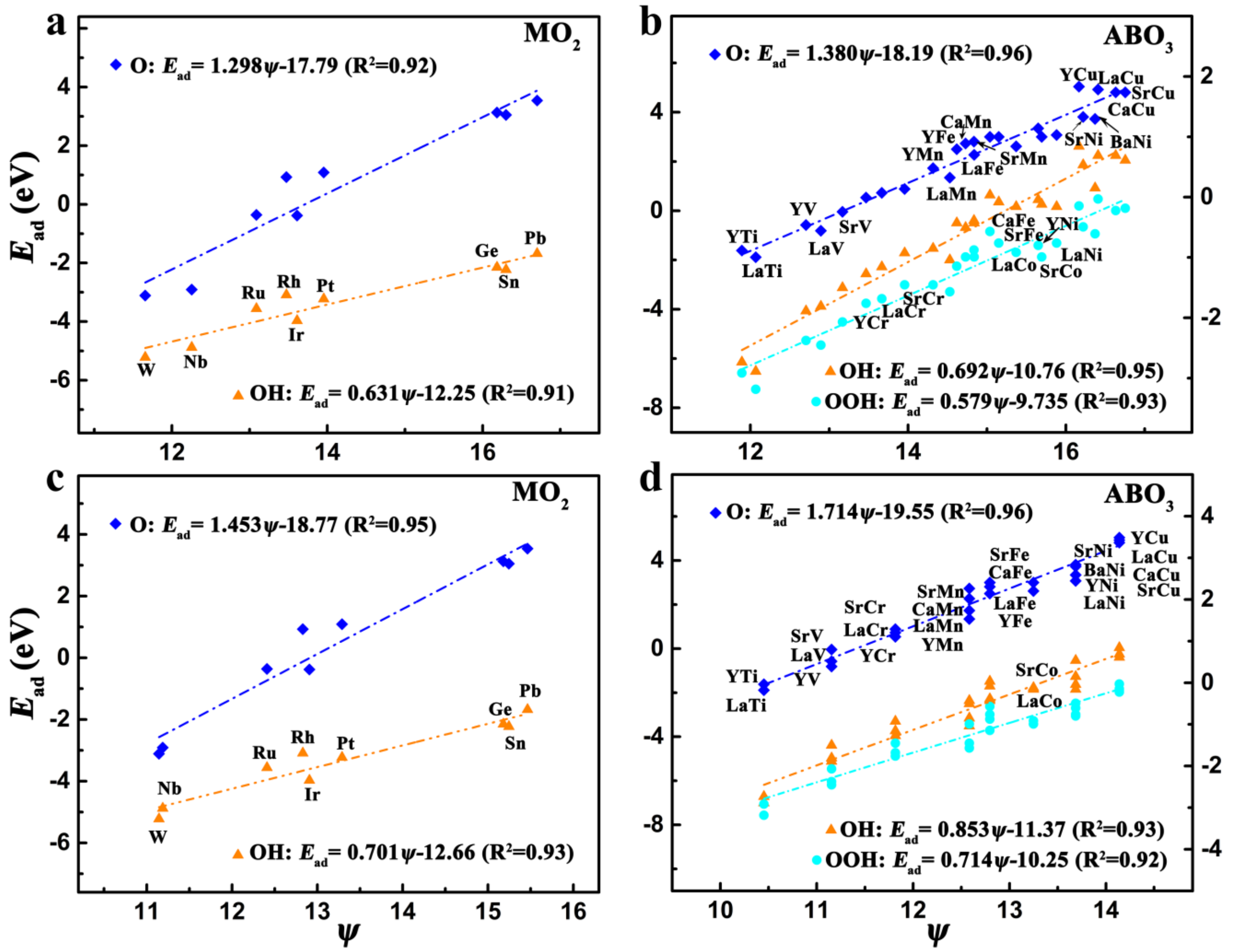

Supplementary Figure 11 | Effect of the second-nearest neighbors on the scaling relation between the electronic descriptor ψ and adsorption energy. The adsorption energies of O, OH and OOH on (a) [(c)] (110) surface of dioxides MO_2(110) and (b) [(d)] (100) surface of perovskite oxides ABO_3(100) as a function of ψ including [without] the effect of the second-nearest neighbors[3,5]. In each subfigure with both left and right axes, the linear fits at the upper left corner correspond to the left coordinate axis and those at the bottom right corner correspond to the right coordinate axis. Clearly, the second-nearest neighbors only have a minor effect on the scaling relation between ψ and adsorption energy. Source data are provided as a Source Data file.

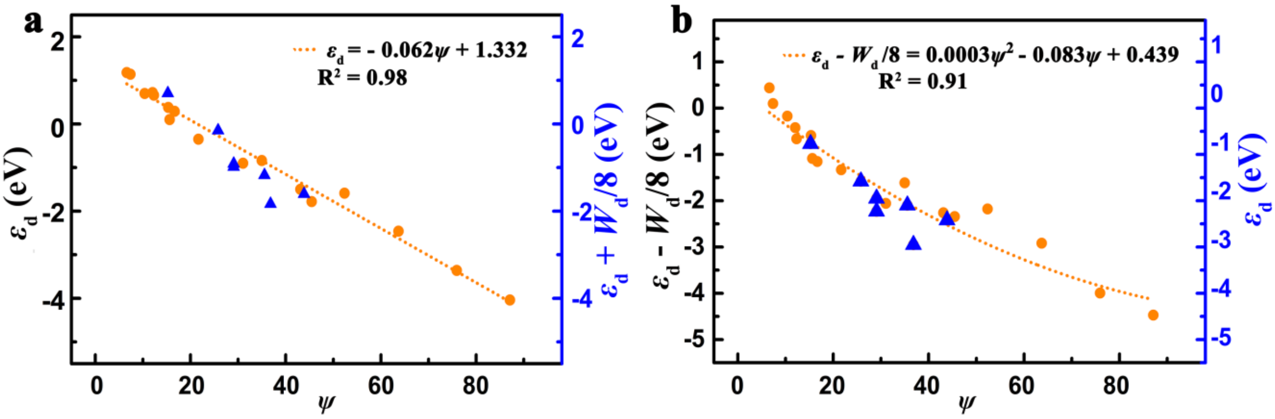

Supplementary Figure 12 | The connection between d-band model and the electronic descriptor *ψ*. (a) The d-band center ε_d of the semi-elliptical distribution of density of state (DOS) versus ψ for the 17 transition metals (TMs), along with the ε_d+W_d/8 (with W_d being the d-band width) of the semi-elliptical distribution of DOS versus ψ for the 7 other TMs[4]. (b) The ε_d-W_d/8 of the semi-elliptical distribution of DOS versus ψ for the 17 TMs, along with the ε_d of the semi-elliptical distribution of DOS versus ψ for the other 7 TMs[4]. Source data are provided as a Source Data file.

Supplementary Table 1. Coordination number matrix for various sites on transition-metal (TM) extended surfaces. AD: adatom, T: terrace, SE: step edge, K: kink.

**site**	$\overline{CN}$
2AD@211	1.667
1AD@111	2.500
2AD@111	2.917
1AD@100	3.000
3AD@111	3.500
2AD@100	3.583
4AD@100	4.417
211 KSE (*CN*=6)	4.667
532 T	4.750
211 SE	5.500
553 SE	5.500
711 SE	5.500
110 SE	5.833
211 KSE (*CN*=8)	6.417
100 T (hollow)	6.615
100 T	6.667
111 T (FCC-hollow)	6.955
111 T	7.500
cavity 111	8.000

Supplementary Table 2. Coordination number matrix for various sites on transition-metal (TM) nanoparticles (NPs) and nanowires (NWs). AD: adatom, T: terrace, E: edge, K: kink, c: center, m: middle, *s*: square, *p*: pentagonal, *h*: hexagonal.

site	$\overline{CN}$	site	$\overline{CN}$
NP_{13} corner	3.500	NP_{181} corner	4.33
NP_{38} 111 E	4.000	NP_{181} 111 E (near corner)	6.00
NP_{38} 111 T	6.000	NP_{181} 111 E	6.08
NP_{44} site-6[23]	5.00	NP_{181} 111 T (c)	7.00
NP_{44} site-5[23]	5.67	NP_{201} 1AD@111 (near 111 E)	2.083
NP_{46} site-4[23]	4.33	NP_{201} 2AD@111 (near 111 E)	2.500
NP_{47} site-6[23]	4.50	NP_{201} 1AD@100	2.670
NP_{49} site-5[23]	4.42	NP_{201} 2AD@100	3.250
NP_{49} site-6[23]	4.92	NP_{201} corner	4.250
NP_{68} K	3.167	NP_{201} 111 E	5.000
NP_{68} 111 E	4.500	NP_{201} 100 E	5.167
NP_{68} 111 T (m)	6.500	NP_{201} 100 T (c)	6.333
NP_{68} 111 (c)	7.500	NP_{201} 111 T (m)	6.917
NP_{79} K	4.083	NP_{201} 111 T (c)	7.500
NP_{79} 111 E	5.000	NP_{586} 111 T (c)	7.500
NP_{79} 111 T	6.667	NP_{309} corner	4.33
NP_{120} 111 E	3.25	NP_{309} 111 E	6.33
NP_{120} 111 T	6.00	NP_{309} facet	7.33
NP_{147} K	3.333	NP_{365} corner	2
NP_{147} corner	4.333	NP_{365} 100 E	3.67
NP_{147} 100 E	5.000	NP (~2 nm) corner	3.33
NP_{147} 100 T (c)	6.500	NP (~2 nm) 100 E	5.33
NP_{147} 111 T (c)	6.500	*s*-NW 100 E	3.67
NP_{171} corner	3.17	*p*-NW 100 E	4.33
NP_{171} 100 E	3.67	*h*-NW 111 E	5.17
NP_{171} 111 T (m)	5.83	*h*-NW 100 E	5.33
NP_{171} 111 T (c)	6.67		

Supplementary Table 3. Summary of valence-electron number S_v, Pauling electronegativity χ, and descriptor ψ for transition metals in periods 4-6 of the periodic table of elements.

Element	S_v	χ	ψ
Sc	3	1.36	6.62
Ti	4	1.54	10.39
V	5	1.63	15.34
Cr	6	1.66	21.69
Mn	7	1.55	31.61
Fe	8	1.83	34.97
Co	9	1.88	43.09
Ni	10	1.91	52.36
Cu	11	1.90	63.68
Zn	12	1.65	87.27
Y	3	1.22	7.38
Zr	4	1.33	12.03
Nb	5	1.60	15.63
Mo	6	1.16	31.03
Tc	7	1.90	25.79
Ru	8	2.20	29.09
Rh	9	2.28	35.53
Pd	10	2.20	45.45
Ag	11	1.93	87.10
Cd	12	1.69	85.21
La	3	1.10	8.18
Hf	4	1.30	12.31
Ta	5	1.50	16.67
W	6	2.36	15.25
Re	7	1.90	25.79
Os	8	2.20	29.09
Ir	9	2.20	36.82
Pt	10	2.28	43.86
Au	11	2.54	75.92
Hg	12	2.00	72

Supplementary Table 4. Comparison between the predicted slopes *k* by the established relation and the fitted DFT-calculated ones for different adsorbates on pure transition-metal (TM) close-packed surfaces (CPS) and stepped surfaces (SS). Columns 2 and 3 show the predicted results and the ones derived from data by the PBE+TSsurf method, respectively. Additionally, Columns i-v show the fitted slopes *k* from Refs [1, 3, 5, 14, 28] shown in Fig. 1 in the main text.

**Species**	***k***								
	Predicted	PBE+TSsurf	i[14]		ii[28]		iii[5]	iv[3]	v[1]
		CPS	CPS	SS	CPS	SS	CPS	CPS	SS
C	0.08	——	0.081	0.083	——	——	——	——	——
CH	0.06	——	0.065	0.061	——	——	——	——	——
CH_2	0.04	——	0.040	0.039	——	——	——	——	——
CH_3	0.02	——	0.018	0.020	——	——	——	——	——
CO	0.04	0.042	0.041	0.041	——	——	——	——	——
COH	0.067	——	0.067	0.066	——	——	——	——	——
CHO	0.03	——	0.031	0.035	——	——	——	——	——
CHOH	0.047	——	0.046	0.045	——	——	——	——	——
COOH	0.027	0.031	0.023	0.028	——	——	——	——	——
N	0.075	——	——	——	0.078	0.085	——	——	——
NH	0.05	——	——	——	0.058	0.057	——	——	——
NH_2	0.025	——	——	——	0.033	0.032	——	——	——
NNH_2	0.05	——	——	——	0.057	0.047	——	——	——
O	0.067	——	——	——	——	——	0.075	——	0.061
OH	0.033	——	——	——	——	——	0.035	——	0.031
OOH	0.033	——	——	——	——	——	——	0.040	——
OCH_3	0.033	——	——	——	——	——	——	——	——

Supplementary Table 5. Comparison between the predicted slopes *k* by the established relation and the fitted DFT-calculated ones for different adsorbates on transition-metal (TM) close-packed surfaces (CPS) and stepped surfaces (SS). Column 2 shows the predicted ones while Columns i-v show the fitted results from Refs [1, 12, 14-16] shown in Supplementary Figures 1-4.

Species	***k***							
	Predicted	i[1]		ii[12]		iii[14]	iv[15]	v[16]
		CPS	SS	CPS	SS	(100)	CPS	CPS
C	0.08	0.075	0.081	——	——	0.088	——	——
CH	0.06	0.057	0.062	0.071	0.063	0.065	——	——
CH_2	0.04	0.037	0.042	0.043	0.042	0.041	——	——
CH_3	0.02	0.019	0.021	0.020	0.021	0.018	——	——
CO	0.04	——	——	0.037	0.039	0.040	——	——
COH	0.067	——	——	0.072	0.062	0.067	——	——
CHO	0.03	——	——	0.032	0.036	0.038	——	——
CHOH	0.047	——	——	0.047	0.046	0.043	——	——
COOH	0.027	——	——	——	——	0.024	——	——
N	0.075	——	——	——	——	——	——	——
NH	0.05	——	——	——	——	——	——	——
NH_2	0.025	——	——	——	——	——	——	——
NNH_2	0.05	——	——	——	——	——	——	——
O	0.067	——	——	——	——	——	0.070	0.066
OH	0.033	——	——	——	——	——	0.031	——
OOH	0.033	——	——	——	——	——	——	——
OCH_3	0.033	——	——	——	——	——	——	——

Supplementary Table 6. Comparison between the predicted slopes *k* by the established relation and the fitted DFT-calculated ones for different adsorbates on transition-metal (TM) close-packed surfaces (CPS) and stepped surfaces. Column 2 shows the predicted results while Columns i-vi show the fitted ones from Refs [1, 4, 5, 17-19] shown in Supplementary Figure 4.

Species	***k***										
	Predicted	i[5]			ii[17]	iii[19]		iv[4]	v[1]		vi[18]
		Top site	Bridge site	Fully relaxed	(211)	(110)	(211)	CPS	CPS	(100)	(211)
O	0.067	0.066	0.068	——	0.064	0.063	0.071	0.079	0.067	0.061	0.063
OH	0.033	——	——	0.034	0.032	——	——	——	0.030	0.028	0.033
OOH	0.033	——	——	——	——	——	——	——	——	——	——
OCH_3	0.033	——	——	——	——	——	——	——	——	——	——

Supplementary Table 7. The sum of facets information for various transition-metal (TM) extended surfaces, nanoparticles (NPs) and nanowires (NWs) in Figs 2 and 3 in the main text. AD: adatom, T: terrace, SE: step edge, K: kink, E: edge, c: center, m: middle, *s*: square, *p*: pentagonal, *h*: hexagonal.

**Fig. 2a**		**Fig. 2b**	**Fig. 2c**
CO & COOH	CHO & COH	cavity 111	111 T
Au_{309} corner	111 T (FCC-hollow)	111 T	110 SE
Au_{309} 111 E	100 T (hollow)	100 T	100 T
Au_{365} corner	211 SE	110 SE	211 SE
Au_{365} 100 E	211 KSE (*CN*=6)	711 SE	Ag_{13} corner
Au_{NP} (~2 nm) corner	3AD@111	211SE	Ag_{147} corner
Au_{NP} (~2 nm) 100 E		211 KSE (*CN*=6)	Ag_{309} corner
s-NW 100 E		4AD@100	Ag_{309} facet
p-NW 100 E		3AD@111	
h-NW 111 E		2AD@111	
h-NW 100 E			
111 T			
100 T			
110 SE			
211 SE			
**Fig. 2d**	**Fig. 3a**		**Fig. 3b-e**
2AD@111	2AD@211	Pt_{68} 111 (c)	111 T
2AD@100	1AD@111	Pt_{79} K	100 T
4AD@100	2AD@111	Pt_{79} 111 E	211 KSE (*CN*=8)
711 SE	1AD@100	Pt_{79} 111 T	553 SE
533 SE	3AD@111	Pt_{147} K	110 SE
100 T	2AD@100	Pt_{147} 100 E	211 SE
111 T	4AD@100	Pt_{147} 100 T (c)	211 KSE (*CN*=6)
	211 KSE (*CN*=6)	Pt_{147} 111T (c)	4AD@100
	211 SE	Pt_{201} 1AD@111	3AD@111
	553 SE	Pt_{201} 2AD@111	2AD@100
	110 SE	Pt_{201} 1AD@100	2AD@111
	211 KSE (*CN*=8)	Pt_{201} 2AD@100	2AD@211
	100 T	Pt_{201} corner	
	111 T	Pt_{201} 111 E	
	Pt_{38} 111 E	Pt_{201} 100 E	
	Pt_{38} 111 T	Pt_{201} 100 T (c)	
	Pt_{68} K	Pt_{201} 111 T (m)	
	Pt_{68} 111 E	Pt_{201} 111 T (c)	
	Pt_{68} 111 T(m)	Pt_{586} 111T (c)	

Supplementary Table 8. Comparison between the predicted prefactors λ by the established relation and the fitted DFT-calculated ones for different adsorbates on transition-metal (TM) surfaces and nanoparticles. Column 2 shows the predicted results while Columns i-x show the fitted ones from Refs [20, 22-24, 29-32] shown in Fig. 2 in the main text and Supplementary Figure 5.

**Species**	$\boldsymbol{\lambda}$										
	Predicted	i[29]	ii[30]	iii[31]	iv[32]	v[20]	vi[22]	vii[23]	viii[24]	ix[24]	x[24]
		Au	Cu	Ag	Pt	Au	Au	Au	Cu	Ni	Au
C	0.04	——	——	——	——	——	——	——	——	——	——
CH	0.08	——	——	——	——	——	——	——	——	——	——
CH_2	0.12	——	——	——	——	——	——	——	——	——	——
CH_3	0.16	——	——	——	——	——	——	——	——	——	——
CO	0.12	0.133	0.116	0.097	——	0.132	0.163	0.158	——	——	0.145
COH	0.067	——	——	——	——	——	——	——	——	——	0.074
CHO	0.14	——	0.127	——	——	0.127	——	——	0.119	0.144	0.112
CHOH	0.106	——	——	——	——	——	——	——	——	——	——
COOH	0.147	0.125	0.158	0.154	——	0.142	——	——	——	——	——
N	0.05	——	——	——	0.066	——	——	——	——	——	——
NH	0.1	——	——	——	0.111	——	——	——	——	——	——
NH_2	0.15	——	——	——	——	——	——	——	——	——	——
NNH_2	0.1	——	——	——	——	——	——	——	——	——	——

Supplementary Table 9. Comparison between the predicted prefactors λ by the established relation and the fitted DFT-calculated ones for different adsorbates on transition-metal (TM) surfaces and nanoparticles. Column 2 shows the predicted results while Columns i-vii show the fitted ones from Refs [2, 21, 25] shown in Fig. 3 in the main text and Supplementary Figure 5.

**Species**	$\boldsymbol{\lambda}$							
	Predicted	i[2]	ii[2]	iii[2]	iv[2]	v[25]	vi[25]	vii[21]
		Au	Co	Cu	Ni	Pt	Au	Pt
O	0.067	——	0.088	——	0.077	——	——	——
OH	0.133	0.155	0.145	0.101	0.113	0.192	0.128	0.184
OOH	0.133	0.114	0.148	0.103	0.104	0.169	0.141	0.154
OCH_3	0.133	0.157	0.143	0.089	0.124	——	——	——

Supplementary Table 10. Summary of descriptor ψ for near-surface alloys (NSAs) Pd-X@Pd(100), Pt-X@Pt(111) and Pt-X@Pt(100). NSAs effectively fills in the gaps of ψ between different transition metals (TMs), suggesting a great potential of alloying in engineering the adsorption energy.

**Pd-X@Pd**		**Pt-X@Pt**			
**(100)**	ψ	**(100)**	ψ	**(111)**	ψ
Sc	19.30	Sc	18.92	Ti	28.47
Ti	23.59	Ti	23.12	V	32.00
V	28.05	V	27.50	Cr	35.51
Y	20.26	Y	19.86	Mn	39.76
Zr	25.18	Zr	24.68	Fe	40.98
Nb	28.28	Nb	27.72	Co	43.63
Ru	37.28	Ru	36.54	Ni	46.25
Rh	40.74	Rh	39.94	——	——
Pd	45.45	Pd	44.56	——	——
La	21.21	La	20.80	——	——
Ta	29.10	Ta	28.53	——	——
W	27.98	W	27.43	——	——
——	——	Re	34.64	——	——
Os	37.28	Os	36.54	——	——
Ir	41.39	Ir	40.58	——	——
Pt	44.74	Pt	43.86	——	——
Au	46.41	Au	45.5	——	——

Supplementary Table 11. Summary of descriptor ψ for monoxides MO(100), dioxides MO_2(110) and perovskite oxides ABO_3(100). It is noteworthy that when modulating ψ, metallic materials and oxides are very close in altering the adsorption energy.

**MO(100)**	ψ	**MO_2(110)**	ψ	**ABO_3(100)**	ψ
CaO	8.92	GeO_2	16.18	$LaTiO_3$	12.07
ScO	9.70	IrO_2	13.61	$LaVO_3$	12.90
TiO	10.45	NbO_2	12.26	$LaCrO_3$	13.67
VO	11.15	PbO_2	16.70	$LaMnO_3$	14.53
CrO	11.82	PtO_2	13.95	$LaFeO_3$	14.83
MnO	12.58	RhO_2	13.47	$LaCoO_3$	15.37
FeO	12.80	RuO_2	13.09	$LaNiO_3$	15.88
CoO	13.25	SnO_2	16.30	$LaCuO_3$	16.41
NiO	13.69	WO_2	11.65	$YTiO_3$	11.89
CuO	14.14	——	——	YVO_3	12.71
——	——	——	——	$YCrO_3$	13.47
——	——	——	——	$YMnO_3$	14.32
——	——	——	——	$YFeO_3$	14.62
——	——	——	——	$YNiO_3$	15.65
——	——	——	——	$YCuO_3$	16.17
——	——	——	——	$SrVO_3$	13.17
——	——	——	——	$SrCrO_3$	13.96
——	——	——	——	$SrMnO_3$	14.84
——	——	——	——	$SrFeO_3$	15.15
——	——	——	——	$SrCoO_3$	15.69
——	——	——	——	$SrNiO_3$	16.22
——	——	——	——	$SrCuO_3$	16.76
——	——	——	——	$CaMnO_3$	14.73
——	——	——	——	$CaFeO_3$	15.04
——	——	——	——	$CaCuO_3$	16.63
——	——	——	——	$BaNiO_3$	16.37

Supplementary Table 12. Comparison between the predicted slopes *k* by the established relation and the fitted DFT-calculated ones for different adsorbates on near-surface alloy (NSA) surfaces. Column 2 shows the predicted results, while Columns i-iv show the fitted ones from Refs [6, 26, 33, 34] and Ref. [26] shown in Fig. 4 in the main text and Supplementary Figure 7. Note that all of the slopes *k* here are absolute values.

**Species**	***k***					
	Predicted	i[33]		ii[6]	iii[34]	iv[26]
		Pt	Pd	Pt	Pt	Pt
C	0.08	——	——	0.083	——	0.080
CH	0.06	——	——	0.082	——	——
CH_2	0.04	——	——	0.036	——	——
CH_3	0.02	——	——	0.026	——	——
CO	0.04	0.039	0.034	0.032	——	——
N	0.075	——	——	0.073	——	0.078
NH	0.05	——	——	0.063	——	——
NH_2	0.025	——	——	0.022	——	——
O	0.067	——	——	——	——	0.068
OH	0.033	——	——	——	0.047	——
F	0.050	——	——	——	——	0.053
Cl	0.050	——	——	——	——	0.049
P	0.075	——	——	——	——	0.074

Supplementary Table 13. Comparison between the predicted slopes *k* by the established relation and the fitted DFT-calculated ones for different adsorbates on surfaces of oxides. Column 2 shows the predicted results, while Columns i-iii show the fitted ones from Refs [3, 5] shown in Fig. 5 in the main text, and Columns iv-v correspond to the fitted results from Refs [16, 27] shown in Supplementary Figure 8.

**Species**	***k***					
	Predicted	i[3]	iii[3]	ii[5]	iv[27]	v[16]
O	1.334	1.371	1.380	1.298	1.487	1.358
OH	0.667	0.695	0.692	0.631	0.873	0.767
OOH	0.667	0.590	0.579	——	——	——
OCH_3	0.667	——	——	——	0.795	——

Supplementary Table 14. The predicted several nanoparticles (NPs) and near-surface alloys (NSAs) systems that have similar adsorption energies with respect to the cases of CO on Cu(111) and OH on Pt(111), respectively.

**Adsorbates**	**Substrates**	**Predicted substrates**	
		NPs	NSAs
CO	Cu(111)	Au_{13} corner	Pd-La@Pd(100)
		Au_{147} kink	Pd-Ti@Pd(100)
		——	Pd-Sc@Pd(100)
		——	Pd-Y@Pd(100)
OH	Pt(111)	Pd_{201} terrace (m)	Pt-Mn@Pt(111)
		Pd_{181} terrace (c)	Pt-Fe@Pt(111)
		——	Pt-Co@Pt(111)
		——	Pt-Mo@Pt(111)
		——	Pt-Rh@Pt(111)
		——	Pt-Pd@Pt(111)
		——	Pt-Ir@Pt(111)

Supplementary Table 15. Comparison between the predicted and DFT-calculated prefactors (ψ and $\overline{CN}$) for 5 reaction steps of CO_2RR on transition-metal (TM) surfaces. The DFT-calculated results are extracted from Ref. [14].

**Reaction steps**	**Prefactors of ψ**		**Prefactors of $\overline{CN}$**	
	**Predicted**	**Calculated**	**Predicted**	**Calculated**
CO→COH	-0.027	-0.011	0.054	——
COH→C	-0.013	-0.012	0.026	——
CO→CHO	0.010	0.008	-0.020	——
COOH→CO	-0.013	-0.010	0.026	——
CH→CH_2	0.020	0.019	-0.040	——

Supplementary Table 16. Coordination number matrix for top site on surfaces of near-surface alloys (NSAs), oxides and perovskite oxides.

**NSAs**	$\boldsymbol{N_{adsorption}}$ **(A)**	$\boldsymbol{N_{1st}}$ **(A)**	$\boldsymbol{N_{1st}}$ **(B)**	**-**
A-B@A(100)	1	4	4	-
A-B@A(111)	1	6	3	-
**Oxides**	$\boldsymbol{N_{adsorption}}$ **(M)**	**N_{1st} (O)**		**N_{2nd} (M)**
MO(100)	1	5		-
MO_2(110)	1	5		1
**Perovskite oxides**	$\boldsymbol{N_{adsorption}}$ **(B)**	**N_{1st} (O)**		**N_{2nd} (A)**
ABO_3(100)	1	5		1

Supplementary Table 17. Summary of the gas references for the data cited by the study. For hydrogenates $AH_{x_{max}}$, the central atom A could be C, O, N and S, while x_{max} is the maximum number that the central atom can bond to hydrogen.

Title	Gas references
Trends in electrochemical CO_2 reduction activity for open and close-packed metal surfaces[14]	CO, H_2O, H_2
A theoretical evaluation of possible transition metal electro-catalysts for N_2 reduction[28]	N_2, H_2
An electronic structure descriptor for oxygen reactivity at metal and metal oxide surfaces[5]	O_2, H_2
Machine-learning-augmented chemisorption model for CO_2 electroreduction catalyst screening[33]	CO, H_2, H_2O
Electronic structure engineering in heterogeneous catalysis: identifying novel alloy catalysts based on rapid screening for materials with desired electronic properties[6]	CO, H_2, H_2O
Predictive structure reactivity models for rapid screening of Pt-based multimetallic electrocatalysts for the oxygen reduction reaction[34]	
Physical and chemical nature of the scaling relations between adsorption energies of atoms on metal surfaces[26]	Single atom
Accounting for bifurcating pathways in the screening for CO_2 reduction catalysts[24]	CO, H_2
Understanding the effects of Au morphology on CO_2 electrocatalysis[29]	Graphene, H_2, H_2O
	CO
Generalized surface coordination number as an activity descriptor for CO_2 reduction on Cu surfaces[30]	Graphene, H_2, H_2O
Active sites of Au and Ag nanoparticle catalysts for CO_2 electroreduction to CO[31]	Graphene, H_2, H_2O
	CO
Bond-making and breaking between carbon, nitrogen, and oxygen in electrocatalysis[32]	N_2, H_2
Finding optimal surface sites on heterogeneous catalysts by counting nearest neighbors[25]	H_2O, H_2
Introducing structural sensitivity into adsorption–energy scaling relations by means of coordination numbers[2]	O_2, OH, OOH, OCH_3

Title	Gas references
Understanding trends in electrochemical carbon dioxide reduction rates[12]	CO, H_2, H_2O
Origin of the overpotential for oxygen reduction at a fuel-cell cathode[15]	H_2O, H_2
Scaling relationships for adsorption energies on transition metal oxide, sulfide, and nitride surfaces[16]	$AH_{x_{max}}$, H_2
Using scaling relations to understand trends in the catalytic activity of transition metals[17]	$AH_{x_{max}}$, H_2
Theoretical trends in particle size effects for the oxygen reduction reaction[18]	H_2O, H_2
Electronic structure effects in transition metal surface chemistry[4]	O_2
Descriptors and thermodynamic limitations of electrocatalytic carbon dioxide reduction on rutile oxide surfaces[27]	CO_2, H_2, H_2O
Physical and chemical nature of the scaling relations between adsorption energies of atoms on metal surfaces[26]	Single atom
Tuning the selectivity and activity of Au catalysts for carbon dioxide electroreduction via grain boundary engineering: a DFT study[20]	Graphene, H_2, H_2O
	CO
Orbitalwise coordination number for predicting adsorption properties of metal nanocatalysts[22]	CO, O_2
Assessment of catalytic activities of gold nanoclusters with simple structure descriptors[23]	CO, O_2

Supplementary Table 18. Summary of the standard gas references in this study.

*Adsorbates	Standard gas references
C, CH_x (x=0~3), CO, COH, CHO, COOH	CO, H_2O, H_2
N, NH_x (x=0~2), NNH_2	N_2, H_2
O	$1/2O_2$
OH	OH
OOH	OOH
OCH_3	OCH_3

Supplementary References

1. Abild-Pedersen, F. *et al.* Scaling properties of adsorption energies for hydrogen-containing molecules on transition-metal surfaces. *Phys. Rev. Lett.* **99**, 016105 (2007).
2. Calle-Vallejo, F., Loffreda, D., Koper, M. T. M. & Sautet, P. Introducing structural sensitivity into adsorption-energy scaling relations by means of coordination numbers. *Nat. Chem.* **7**, 403–410 (2015).
3. Calle-Vallejo, F. *et al.* Number of outer electrons as descriptor for adsorption processes on transition metals and their oxides. *Chem. Sci.* **4**, 1245–1249 (2013).
4. Vojvodic, A., Nørskov, J. K. & Abild-Pedersen, F. Electronic structure effects in transition metal surface chemistry. *Top. Catal*. **57**, 25–32 (2014).
5. Dickens, C. F., Montoya, J. H., Kulkarni, A. R., Bajdich, M. & Nørskov, J. K. An electronic structure descriptor for oxygen reactivity at metal and metal-oxide surfaces. *Surf. Sci.* **681**, 122–129 (2019).
6. Xin, H., Holewinski, A., Schweitzer, N., Nikolla, E. & Linic, S. Electronic structure engineering in heterogeneous catalysis: identifying novel alloy catalysts based on rapid screening for materials with desired electronic properties. *Top. Catal*. **55**, 376–390 (2012).
7. Montoya, J. H., Doyle, A. D., Nørskov, J. K. & Vojvodic, A. Trends in adsorption of electrocatalytic water splitting intermediates on cubic ABO_3 oxides. *Phys. Chem. Chem. Phys.* **20**, 3813–3818 (2018).
8. Peterson, A. A., Abild-Pedersen, F., Studt, F., Rossmeisl, J. & Nørskov, J. K. How copper catalyzes the electroreduction of carbon dioxide into hydrocarbon fuels. *Energy Environ. Sci.* **3**, 1311 (2010).
9. Perdew, J. P., Burke, K. & Ernzerhof, M. Generalized gradient approximation made simple. *Phys. Rev. Lett.* **77**, 3865–3868 (1996).
10. Kresse, G. & Furthmüller, J. Efficient iterative schemes for ab initio total-energy calculations using a plane-wave basis set. *Phys. Rev. B* **54**, 11169–11186 (1996).
11. Hammer, B., Hansen, L. B. & Nørskov, J. K. Improved adsorption energetics within density-functional theory using revised Perdew-Burke-Ernzerhof functionals. *Phys. Rev. B* **59**, 7413–7421 (1999).
12. Liu, X. *et al.* Understanding trends in electrochemical carbon dioxide reduction rates. *Nat. Commun.* **8**, 15438 (2017).
13. Wellendorff, J. *et al.* Density functionals for surface science: exchange-correlation model development with Bayesian error estimation. *Phys. Rev. B* **85**, 235149 (2012).
14. Shi, C., Hansen, H. A., Lausche, A. C. & Nørskov, J. K. Trends in electrochemical CO_2 reduction activity for open and close-packed metal surfaces. *Phys. Chem. Chem. Phys.* **16**, 4720-4727 (2014).
15. Nørskov, J. K. *et al.* Origin of the overpotential for oxygen reduction at a fuel-cell cathode. *J. Phys. Chem. B* **108**, 17886–17892 (2004).
16. Fernndez, E. M. *et al.* Scaling relationships for adsorption energies on transition metal oxide, sulfide, and nitride surfaces. *Angew. Chem. Int. Ed.* **47**, 4683–4686 (2008).
17. Jones, G., Bligaard, T., Abild-Pedersen, F. & Nørskov, J. K. Using scaling relations to understand trends in the catalytic activity of transition metals. *J. Phys. Condens. Matter* **20**, 064239 (2008).
18. Greeley, J., Rossmeisl, J., Hellmann, A. & Norskov, J. K. Theoretical trends in particle size effects for the oxygen reduction reaction. *Z. Phys. Chem.* **221**, 1209–1220 (2007).
19. Hummelshøj, J. S., Abild-Pedersen, F., Studt, F., Bligaard, T. & Nørskov, J. K. CatApp: a web application for surface chemistry and heterogeneous catalysis. *Angew. Chem. Int. Ed.* **51**, 272–274 (2012).
20. Dong, C. *et al.* Tuning the selectivity and activity of Au catalysts for carbon dioxide electroreduction via grain boundary engineering: a DFT study. *J. Mater. Chem. A* **5**, 7184–7190 (2017).
21. Calle-Vallejo, F., Martínez, J. I., García-Lastra, J. M., Sautet, P. & Loffreda, D. Fast prediction of adsorption properties for platinum nanocatalysts with generalized coordination numbers. *Angew. Chem. Int. Ed.* **53**, 8316–8319 (2014).
22. Ma, X. & Xin, H. Orbitalwise coordination number for predicting adsorption properties of metal nanocatalysts.

Phys. Rev. Lett. **118**, 036101 (2017).

23. Xu, H., Cheng, D., Gao, Y. & Zeng, X. C. Assessment of catalytic activities of gold nanoclusters with simple structure descriptors. *ACS Catal.* **8**, 9702–9710 (2018).
24. Calle-Vallejo, F. & Koper, M. T. M. Accounting for bifurcating pathways in the screening for CO_2 reduction catalysts. *ACS Catal.* **7**, 7346–7351 (2017).
25. Calle-Vallejo, F. *et al.* Finding optimal surface sites on heterogeneous catalysts by counting nearest neighbors. *Science* **350**, 185–189 (2015).
26. Calle-Vallejo, F., Martínez, J. I., García-Lastra, J. M., Rossmeisl, J. & Koper, M. T. M. Physical and chemical nature of the scaling relations between adsorption energies of atoms on metal surfaces. *Phys. Rev. Lett.* **108**, 116103 (2012).
27. Bhowmik, A., Vegge, T. & Hansen, H. A. Descriptors and thermodynamic limitations of electrocatalytic carbon dioxide reduction on rutile oxide surfaces. *ChemSusChem* **9**, 3230–3243 (2016).
28. Skúlason, E. *et al.* A theoretical evaluation of possible transition metal electro-catalysts for N_2 reduction. *Phys. Chem. Chem. Phys.* **14**, 1235–1245 (2012).
29. Back, S., Yeom, M. S. & Jung, Y. Understanding the effects of Au morphology on CO_2 electrocatalysis. *J. Phys. Chem. C* **122**, 4274–4280 (2018).
30. Zhao, Z., Chen, Z., Zhang, X. & Lu, G. Generalized surface coordination number as an activity descriptor for CO_2 reduction on Cu surfaces. *J. Phys. Chem. C* **120**, 28125–28130 (2016).
31. Back, S., Yeom, M. S. & Jung, Y. Active sites of Au and Ag nanoparticle catalysts for CO_2 electroreduction to CO. *ACS Catal.* **5**, 5089–5096 (2015).
32. Li, H., Li, Y., Koper, M. T. M. & Calle-Vallejo, F. Bond-making and breaking between carbon, nitrogen, and oxygen in electrocatalysis. *J. Am. Chem. Soc.* **136**, 15694–15701 (2014).
33. Ma, X., Li, Z., Achenie, L. E. K. & Xin, H. Machine-learning-augmented chemisorption model for CO_2 electroreduction catalyst screening. *J. Phys. Chem. Lett.* **6**, 3528–3533 (2015).
34. Xin, H., Holewinski, A. & Linic, S. Predictive structure–reactivity models for rapid screening of Pt-based multimetallic electrocatalysts for the oxygen reduction reaction. *ACS Catal.* **2**, 12–16 (2012).
